# Family violence, war, and natural disasters: A study of the effect of extreme stress on children's mental health in Sri Lanka

**DOI:** 10.1186/1471-244X-8-33

**Published:** 2008-05-02

**Authors:** Claudia Catani, Nadja Jacob, Elisabeth Schauer, Mahendran Kohila, Frank Neuner

**Affiliations:** 1 Department of Psychology, University of Konstanz and Center for Psychiatry Reichenau, D-78457 Konstanz, Germany; 2 vivo, Casella Postale no.17, Castelplanio Stazione, I-60032 Ancona, Italy; 3 Vallikamam Educational Zonal Office, Sri Lanka

## Abstract

**Background:**

The consequences of war violence and natural disasters on the mental health of children as well as on family dynamics remain poorly understood. Aim of the present investigation was to establish the prevalence and predictors of traumatic stress related to war, family violence and the recent Tsunami experience in children living in a region affected by a long-lasting violent conflict. In addition, the study looked at whether higher levels of war violence would be related to higher levels of violence within the family and whether this would result in higher rates of psychological problems in the affected children.

**Methods:**

296 Tamil school children in Sri Lanka's North-Eastern provinces were randomly selected for the survey. Diagnostic interviews were carried out by extensively trained local Master level counselors. PTSD symptoms were established by means of a validated Tamil version of the UCLA PTSD Index. Additionally, participants completed a detailed checklist of event types related to organized and family violence.

**Results:**

82.4% of the children had experienced at least one war-related event. 95.6% reported at least one aversive experience out of the family violence spectrum. The consequences are reflected in a 30.4% PTSD and a 19.6% Major Depression prevalence. Linear regression analyses showed that fathers' alcohol intake and previous exposure to war were significantly linked to the amount of maltreatment reported by the child. A clear dose-effect relationship between exposure to various stressful experiences and PTSD was found in the examined children.

**Conclusion:**

Data argue for a relationship between war violence and violent behavior inflicted on children in their families. Both of these factors, together with the experience of the recent Tsunami, resulted as significant predictors of PTSD in children, thus highlighting the detrimental effect that the experience of cumulative stress can have on children's mental health.

## Background

The Northeastern provinces of Sri Lanka serve as an example of an extremely devastating human environment. For two decades, thousands of people have been killed and many more have been displaced as a result of conflict and violence in the country. The Liberation Tigers of Tamil Elam (LTTE) has been fighting for political autonomy, which resulted in ongoing conflict. Civilians have become casualties of war; landmines and unexploded ordnance pose a constant threat; hospitals and schools have been destroyed; and the economy has been shattered. A cease-fire agreement that was called in December 2001 lead to a relatively undisturbed period until Sri Lanka was hit by the Asian Tsunami in December 2004. A giant tidal wave destroyed widespread coastal areas throughout Sri Lanka including the North-Eastern provinces. It caused more than 30,000 casualities in Sri Lanka, and hundreds of thousands of inhabitants had to be relocated to relatives' homes and provisional camps. After the disaster, tensions between conflict parties grew and eventually the civil war broke out again, costing the lives of nearly 3,000 people in a year alone [1].

Current armed conflicts are characterized by high levels of deliberate and systematic violence against the civil population that impact society at multiple levels including the individual, family, and community [2]. Studies on the psychological consequences of wars on children have shown increased rates of mental disorders in traumatized children, particularly posttraumatic stress disorder (PTSD). Statistics range from 20% PTSD prevalence among Lebanese children who were exposed to bombings and terror attacks [3] and among refugee children in Germany [4] to a 44% prevalence in surviving orphans 10 years after the Rwandan genocide [5]. Kuwert and co-workers [6] found a persisting high prevalence of war-associated PTSD in adults who have been children during World War II even 60 years after the war. Most recently, a survey of Tamil school children exposed to war in the North-Eastern provinces of Sri Lanka showed that 19–25% of the sample were suffering from PTSD even before the tsunami [7].

Major natural disasters like hurricanes, earthquakes and floods, can also be extremely stressful for the population and cause similar mental health effects as wars [8,9]. Again, children are particularly vulnerable. Depending on the severity of the disaster within the particular region, PTSD rates between 14 and 95% have been found among children living in different communities affected by Hurricane Mitch in Nicaragua [10] and an earthquake-affected region in Armenia [11].

The Northeast region of Sri Lanka has suffered both a long history of civil war and a severe natural disaster, the tsunami. Epidemiological studies in war-affected populations have found a linear relationship between the number of war event types and PTSD symptoms in both children and adults [12,13]. Extending this dose-effect model of war events to a variety of traumatic events beyond war, a high prevalence of PTSD can be expected within the Sri Lankan children who have been affected by additional traumatic events. Indeed, the prevalence of PTSD in Sri Lankan children affected by both the civil war and a natural disaster was as high as 40% after the Tsunami [14]. The major predictor of PTSD was the cumulative trauma exposure including war and disaster events.

Apart from war and Tsunami related traumatic stress, there is reason to expect that additional aversive and stressful events are going to occur within severely affected communities. These additional traumatic events may in turn increase the risk of mental health disorders. Following the so-called "cycle of violence" hypothesis [15,16], one would predict that high levels of war violence lead to higher levels of violence within the family and community. The original assumption of this model suggests that violence is transmitted intergenerationally; hence, a childhood history of physical maltreatment predisposes the survivor to be a perpetrator of violence as an adult, resulting in a continuing cycle of violence and abuse. This hypothesis has been addressed by a number of studies arguing for an intergenerational transmission of abusive behavior at least to some extent [17,18]; however, there is little evidence to support this assumption in war situations. So far, there has been no systematic survey that documents the frequency of family violence or child maltreatment in conflict or post-conflict countries. Some sporadic findings have been published by several organizations and agencies working in refugee camps. These findings suggest that war violence translates into family violence, as evidenced by high levels of spousal beatings in refugee and resettlement communities [19]. There is also some evidence from studies with traumatized Vietnam veterans indicating that traumatized veterans are more prone to engage in domestic abuse [20,21]. In our view, the cycle of violence hypothesis can include wars and organized violence as potential triggers. By investigating whether the experience of war compared to the experience of a natural disaster is related to an increase in family violence we aim at finding empirical support for a contextual extension of the original cycle of violence hypothesis.

Studies within industrialized countries, mainly in the U.S., have identified several risk factors for family violence on children. The main predictors comprise parents' psychopathology, parents' unemployment, poverty and poor nutrition [22]. Another major risk factor is substance abuse, as children living in affected households are more likely to experience abuse or neglect than children in non substance-abusing households [23,24]. There is strong evidence of a link between traumatic war events and increased levels of substance abuse as well as a high comorbidity between PTSD and alcohol abuse [25,26].

In summary, there is evidence to assume that high levels of child maltreatment and violence in war-affected families exist. The aims of the present investigation were thus twofold. First, we wanted to determine the extent and the potential predictive power of cumulative traumatic and stressful experiences related to war, natural disaster and family violence on mental health functioning in children. Second, we predicted that war related violence can lead to higher levels of violence in the affected families thereby arguing for a 'cycle of violence' model that can be applied not only to individual intragenerational violence but also to the broader family and community level. We assessed the frequency and predictors of family violence experienced and witnessed by a sample of school children living in the Point Pedro area of Northeastern Sri Lanka, a region severely affected by both high levels of violence related to war and the recent Tsunami disaster. We predicted a significant influence of previous war exposure on family violence after controlling for age, gender, as well as economic status. By identifying causes and consequences of adverse childhood experiences and trauma-related mental disorders in this unique sample, we hoped to reveal potential mechanisms for understanding and preventing child maltreatment in the aftermath of war trauma affecting entire communities.

## Method

### Sample

This study was designed as a survey of children attending school in the Vadamarachi school district (Point Pedro area) at the Northern tip of Sri Lanka. Point Pedro and the surrounding Northeastern part of Sri Lanka have been exposed to two decades of civil war and, more recently, to the tsunami.

Children from ages 9–15 from 15 different schools in the Vadamarachi educational zone took part in the survey. We aimed at a random selection of schools in the area, however logistical and security constraints caused by severe unrest in the region during the course of the survey prevented the access of several far-reaching schools. Within the schools, participants were randomly selected on the basis of an index including all classes of the respective school with children aged 9–15. For every class, every fifth child on an alphabetically-ordered list was chosen until the number of children to be interviewed in the respective school was completed. Informed consent was obtained from both the child and one of his or her caregivers or parents. Approval for implementation of the survey in Sri Lanka was obtained from the Ethical Review Board of the University of Konstanz, Germany.

#### Sample Characteristics

The interviewed sample consisted of 296 Tamil school children (158 boys, 138 girls) with ages ranging from 9–15 (mean age: 12.2 years). 56.1% of the children were Hindu and 43.9 % Christian. On average, children attended schools for 7.4 years. 6.4% of the sample had lost their fathers and 4.4 % had lost their mothers. The number of people living in a household with the child ranged from 2 to 14 (average household size 6.4; SD= 2.01).

On average, children had 20 weekly meals (SD = 1.78) and, among those, 12 meals (SD = 2.7) with proteins. The mean number of child possessions (toys and clothes) was 18.46 (SD = 9.26). The number of total family possessions, including furniture (table, beds, mats), kitchen utensils, bikes and cars, televisions, and radios, was on average 40.22 (SD = 25.41). A global index of economic status was calculated as the mean of standardized (z-transformed) variables: child possessions, family possessions, meals/week, and meals with protein/week.

### Procedure

Interviews were conducted by a group of 11 former schoolteachers who had previously been trained as "Master Counselors" [27]. The aim of the training was to allow teachers to identify and refer children with psychological problems, and to offer psychotherapy to them. The training curriculum was based on the manual *"Child Mental Health" *which was developed by local experts on mental health [28]. Counselors were provided with detailed instructions on conducting the survey and the sociodemographic and clinical questionnaires with the children. Interviews conducted by local counselors were closely supervised by one or more clinical psychologists from our work group.

### Instruments

#### Sociodemographic and Clinical Questionnaire and Event Lists

Tamil language versions of all instruments were created using translation and blind reverse translation; i.e., a professional counselor translated from English to the respective local language and independent translators performed the reverse translation, blind to the original version. Discrepancies were checked by experts and a final version was derived through extensive consultation with local counselors and a team of translators.

For the questions related to sociodemography and physical health and for the war-related event list, a modified version of the child questionnaire previously employed in an epidemiological survey on school-children in Vanni area of Sri Lanka [7] was used and adapted to the current conditions given in the Point Pedro area. In addition, the interview contained several questions about parents' drug consumption. In particular, for each parent, children were asked whether they had ever seen him or her consuming alcohol, beetle nut or tobacco. Thereupon, children were asked to estimate the frequency of father's and mother's drug consumption for each used drug using the rating alternatives "once a month", "once in a week" or "about 3 to 4 times a week".

To assess tsunami exposure, five questions related to the tsunami experience were adapted from a questionnaire previously used with Tamil child survivors of the Tsunami disaster [14] and added to the interview. For instance, children were asked "Did you see the big wave close by?" and "Were you caught by the wave?" The five questions were answered yes or no. The score for objective tsunami exposure was the number of "yes" answers.

Family violence experienced by children was defined here as being exposed to physical, emotional or sexual abuse as well as witnessing intimate partner violence between parents. For the assessment of adverse childhood experiences at home we created a questionnaire by selecting items about physical, sexual and emotional abuse as well as neglect from two standard checklists for trauma exposure in childhood: the Early Trauma Inventory [29] and the Childhood Trauma Questionnaire [30]. Following an in-depth discussion with local counselors and experts, the most suitable items for Sri Lankan culture were chosen and the wording was adapted.

It is important to note that with respect to both the war and the family violence event lists, items referred to different *types *of events, not to the *number *of events. Given the frequency of repeated events such as bombings or being slapped at home, it would have been impossible to obtain an accurate count of the number of times these events had occurred. Instead, the number of different event types was established by differentiating between war-related events that happened during the 12 months preceding the interview (scored as "last year") and those that happened more than 12 months prior to the interview (scored as "ever"). The family violence questions were aimed at determining if events had happened "last month" or "ever". Thus, it was possible to determine the number of cases in which there was ongoing family violence.

#### Assessment of Trauma and Depression

The University of California at Los Angeles (UCLA) PTSD Index (UPID) for children [31] was used for the assessment of PTSD symptoms. The UPID has proven good psychometric properties and it has been used in a wide variety of cultural settings [32]. In a previous study, this instrument was translated into Tamil following standard principles of instrument translation and it has subsequently been used with Tamil child survivors of the Tsunami [14]. Rather than relying on a cut-off criterion, we established the diagnosis of PTSD according to ful?llment of the DSM-IV criteria assessed through the corresponding items. For this purpose, we added six items related to problems in functioning in different areas of children's life. A previous validation study showed a high agreement in the PTSD diagnosis (79%) between UPID as administered by the counselors and expert interviews [7]. In addition to the PTSD diagnosis, the UPID allows for the assessment of symptom severity based on the frequency of symptoms reported by the child. For each single DSM-IV symptom, its occurrence within the last month is scored on a scale ranging from 0 (none of the time) to 4 (most of the time). Thus, an overall PTSD severity score can be calculated by summing the scores for each question that corresponds to a symptom resulting in a maximum possible score of 68. To screen for Major Depression and Suicidality, sections A and C of the M.I.N.I. Kid version (Mini-International Neuropsychiatric Interview [33] were additionally administered to the children.

## Results

### Drug use in parents and children

Drug use by fathers appeared to be very prevalent in Sri Lanka, with 46.9 % of the children reporting that their fathers used alcohol and 69.6 % reporting use of other types of drugs. Among the other drugs, betel nut (34.1 %), tobacco (27.9 %) and tobacco plus betel nut (37.5%) were most common. In the majority of cases, frequent alcohol use was reported with 59.7 % of children saying their fathers used alcohol more than 3–4 times a week. Only 0.4 % of the children reported that their mothers used alcohol, whereas 48.7% said their mothers used other types of drugs, predominantly betel nut. This use seemed to be infrequent, however, since all children reported that their mothers use these substances less than once a month. In no single case, children reported using alcohol or any other drug themselves.

### Exposure to war related events

Almost half of the interviewed sample (48.6%) had experienced at least one type of event related to war in the last year. The majority of children (82.4%) reported at least one war-related event during their lifetimes. The most frequent event types reported by the children were seeing a dead or mutilated body (43.6%), being close to a combat situation (39.5%), and witnessing shelling or gunfire (33.1%) On average, Tamil children had experienced 2.73 (SD = 2.3) different types of war events in their life.

### Exposure to family violence

Almost all children (95.6 %) reported at least one family violence event type. In 64.2% of the cases, violence in the family was found to be ongoing. On average, children reported to have experienced or witnessed 5.3 (SD = 3.2) different violent event types in their families.

**Figure 1:**
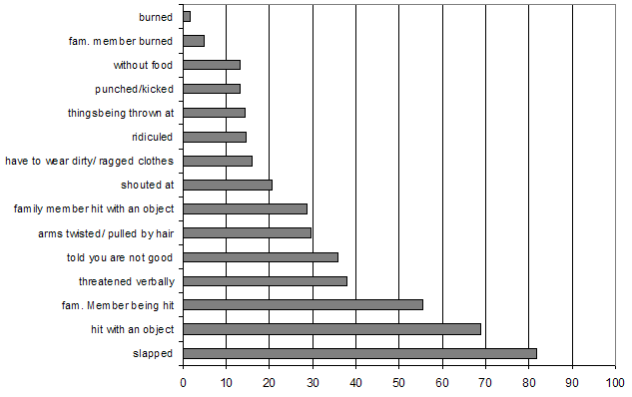
Percentages of children exposed to different incidents of violence at home.

Figure 1 illustrates the frequency of specific types of events described by children again indicating a high level of aggression carried out by the Sri Lankan caregivers. 18% of children had suffered at least one injury because of the violent treatment at home, and 10% of them needed medical treatment on at least one of those occasions. 55.4% of the Sri Lankan sample reported having witnessed other family members being hit, indicating that violence is committed among different members in those families. 4.3% of children reported having experienced or witnessed at least one incident of sexual abuse or sexual violence at home.

### Tsunami Exposure

70.9% of the sample had been directly affected by the Tsunami. Amongst these children, 56.2% had seen the wave close by, 98.6% had to flee from the wave and 22.9 % had been caught by the wave. On average, the Tsunami severity score was 2.4 (SD = 1.28) on a scale ranging from 1 to 5.

### Traumatic Stress and PTSD diagnosis

The analysis of the UPID data revealed that all but one child reported an event that fulfilled the A1 criterion for traumatic event according to the DSM-IV. In 42.3% of the cases, the most upsetting event was related to the Tsunami experience, in another 14.2% it was an incident on the family violence spectrum. 12.8% of the children reported their most upsetting experience as one being related to war. PTSD prevalence was very high with 30.4 % of the children (28.5% of the boys; 32.6% of the girls) fulfilling all DSM-IV criteria for the disorder.

### Prevalence of Major Depressive Disorder and Suicidality

The analysis of the M.I.N.I. data showed that 19.6% of the children met the criteria for a diagnosis of Major Depressive Disorder based on DSM-IV criteria. 22.6% of the children reported past periods of suicidality and 17.2% of the sample was diagnosed with current suicidal ideation.

### Prediction of Family violence

To examine potential predictors for violence experienced by a child at home, we calculated a linear regression model. The dependent variable is the total number of family violence event types reported by the children. Results of the analyses are presented in table 1.

**Table 1:** Prediction of Family Violence: Standardized beta coefficients and zero-order correlation coefficients resulting from a linear regression model on the total amount of family violence experienced

Predictor	β	Zero-order correlation	p-value
Age	-0.04	0.07	0.45
Gender (female)	-0.09	-0.12	0.10
Economic Status	-0.20	-0.09	< 0.001 ***
Father alive (yes)	0.04	0.06	0.50
Mother alive (yes)	0.08	0.08	0.12
Father drinks alcohol (yes)	0.16	0.18	< 0.01 **
Tsunami exposure (yes)	-0.16	-0.14	< 0.01 **
Previous exposure to war (Nr of event types)	0.34	0.38	< 0.001 ***

Note: Full model's adjusted R^2 ^= .22; F(8.27)= 11.11, p < .0001

Zero-order correlation is represented by Spearman's Rho for continuous predictor variables and point-biserial correlation for dichotomous predictor variables

Whereas alcohol use by fathers and the amount of previous war experiences were found as significant positive predictors of family violence, economic status and Tsunami exposure showed a negative relationship with the outcome measure.

### Prediction of PTSD

Predictors of PTSD were established by calculating an additional linear regression model on the UPID symptom severity score (see table 2). The amount of exposure to different types of war events as well as to different forms of family violence together with Tsunami severity resulted as the strongest predictors for PTSD symptom severity. To further investigate the clear relationship between the number of previous stressful and traumatic life events and a current PTSD diagnosis, we divided the whole sample of children into different groups based on the number of stressful event types reported. Aversive experiences related to the war, family violence and the Tsunami were added up to create a global indicator of stressful event load. The initial division was made as follows: the first group consisted of respondents endorsing 0–3 event types, the second group consisted of individuals endorsing 4–7 event types. Each following group endorsed an additional three event types. Because the number of individuals in the groups of 16–19, 20–23, 24–27 and 28–30 event types was very small, these groups were merged to two groups of 16–20 and 21–30 event types.

**Table 2:** Prediction of PTSD symptoms: Standardized beta coefficients and zero-order correlation coefficients resulting from a linear regression model on the UPID symptom severity score

Predictor	β	Zero-order correlation	p-value
Age	-0.05	-0.02	0.37
Gender (female)	0.04	0.05	0.45
Economic status	0.02	-0.12	0.76
Tsunami severity	0.42	0.41	<0.001 ***
Exposure to Family Violence	0.21	0.21	<0.001 ***
Previous exposure to war	0.18	0.26	<0.01 **

Note: Full model's adjusted R^2 ^= .25; F(6.29)= 16.92, p < .0001

Zero-order correlation is represented by Spearman's Rho for continuous predictor variables and point-biserial correlation for dichotomous predictor variables.

**Figure 2:**
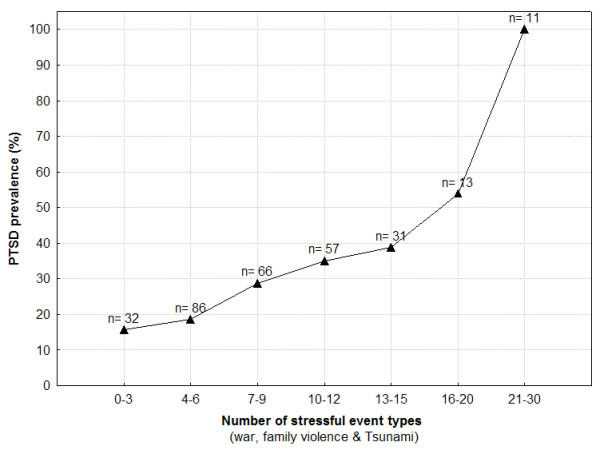
Prevalence of PTSD for children grouped by number of stressful event types.

Figure 2 shows the number of individuals and the prevalence of PTSD in these groups. The number of aversive event types experienced by the children seems to be related to an increased risk of being diagnosed with PTSD. The cumulative trauma threshold in children identified here was 21 events, which means that any child who reported 21 or more different aversive event types was diagnosed with PTSD.

### Outcomes of PTSD

Statistical analyses were conducted to compare children with and without PTSD with respect to somatic complaints and the diagnoses of Major Depressive Disorder and present suicidal ideation. Average values and percentages for each subgroup are listed in table 3. Children with PTSD reported significantly more somatic problems (t = -3.51; p < .001) compared to their peers without PTSD. Furthermore, prevalence of Major Depressive Disorder (Chi-Square = 70.44; p < .001) and present Suicidality (Chi-Square = 47.02; p < .001) was much higher in the group of children diagnosed with PTSD.

**Table 3:** Prevalence of Major Depression, Current Suicidality and Mean Number of Somatic Complaints in children with PTSD and those without the disorder

	Prevalence of Major Depression	Prevalence of current Suicidality	Mean Number of Somatic complaints
Children with PTSD	48.9 % (n = 44)	40 % (n = 36)	2.99 (SD = 1.64)
Children without PTSD	6.8 % (n = 14)	7.3 % (n = 15)	2.28 (SD = 1.49)

## Discussion

One key finding of the present study was the significant relationship between previous war events and the amount of family violence experienced by the children. Together with fathers' alcohol use, exposure to the war resulted as a strong predictor for violence taking place at home. Both violence associated with the war and with parental behavior were in turn related to the diagnosis of PTSD in children. A clear dose-effect relationship between the exposure to different types of stressors (war, Tsunami, and family violence) and PTSD severity was found, suggesting that that the experience of cumulative stress has a harmful effect on children's mental health.

The past and current political situation of Sri Lanka's North East was reflected in the outcomes related to war exposure. More than 80% of the children had experienced at least one war event in the past, and almost half of the sample reported at least one event associated with the violent conflict in the last year.

70.9 % of the sample had been exposed to the Tsunami. The average Tsunami severity score of 2.4 can be regarded as very high, considering that a previous study carried out only four weeks after the Tsunami with Tamil children living in severely affected coastal communities in northeastern Sri Lanka reported comparable rates of exposure [14].

Furthermore, children had experienced or witnessed a mean of 5.2 types of events related to family violence. Physical punishment was found to be very common with 68.8% of the children reporting being beaten with an object. A comparison of data from this study to rates of family violence reported in industrialized countries is difficult as the definition of domestic violence or child maltreatment is still controversial [34,35] and there is a paucity of representative data on child maltreatment prevalence rates [36]. Data from surveys in communities and among child protection services [37] indicate prevalence rates of severe physical abuse between 1 and 5 % in the US. Considering that in the present study 18% of children reported having suffered at least one injury resulting from maltreatment at home, we can conclude that the level of family violence found in the Tamil sample, compared to the numbers reported for industrialized countries, can be regarded as exceptionally high.

Several predictors for children's exposure to family violence emerged from the calculation of a linear regression model. Poverty and parental substance abuse were commonly found as risk factors of child maltreatment in industrialized countries [24,35,38] and could be confirmed as risk factors in our sample.

Given that no systematic survey on family violence inflicted on children in post-conflict countries has been published to date, an essential finding of the present study is the strong relationship between previous war exposure of children and the amount of violence occurring at home. This result gives rise to two possible interpretations. One explanation might be that the parents of the war-affected children in Sri Lanka have experienced more war events themselves. Therefore, the outcome may be in line with previous studies that argue for a link between the traumatizing effects of warfare on men's propensity for domestic abuse [20,21]. Taking into account the potential relationship between war trauma and substance abuse [25], the alcohol intake in fathers, as reported by the children here, could be related to the individual problems associated with war trauma as suggested by the "self-medication" hypothesis [26]. Another interpretation could be that a higher traumatization is related to a higher occurrence of trauma-related symptoms in the affected children–e.g. hyperarousal, irritation, anger outbursts–which in turn causes more frequent acts of punishment by the parents. However, the finding that the Tsunami experience was not predictive of higher levels of family violence, but even inversely related to family violence indicates that the relationship between mass traumas and family violence is more complex. Factors like the duration and nature of mass traumatic event might be crucial for the impact of the events on the family level. One might speculate that the Tsunami as a recent event of short duration has fostered a period of family cohesion whereas the repetitive and chronic stress of war event had a deteriorating effect on family functioning.

Consequences of the extensive amount of stress due to organized as well as family-related violence reported by our sample are reflected in high prevalence rates of psychiatric disorders. About one in three of the children were diagnosed with PTSD and one in five fulfilled the DSM-IV criteria for Major Depression and current suicidal ideation. These findings are in line with previous studies showing increased rates of mental disorders, and particularly PTSD, in children traumatized by violent conflicts [3,5] as well as in child and adolescent victims of natural disasters including the recent Tsunami [10,14]. In this study, we found both the number of aversive experiences related to war and exposure to the Tsunami to be strong predictors for the development of PTSD in the children.

The amount of family violence was identified as an even stronger predictor of PTSD in children than previous exposure to the war. This result highlights the importance that family violence plays in the context of organized violence. This finding is consistent with a number of studies linking different forms of family violence to PTSD and PTSD symptoms [39,40]. Furthermore, there is growing evidence to suggest that being a witness to domestic violence is a stressor severe enough to lead to PTSD in children [41,42].

In the context of severe adversity our data revealed that if the cumulative exposure to stressful events is high enough, any affected child will develop chronic PTSD. Similarly to what has been found for traumatized adults [13], the results support the notion that the repeated occurrence of traumatic stress has a cumulative damaging effect on the mental health of child victims as well as adults.

Consequences of PTSD were found for both physical as well as psychological symptoms. Children with PTSD had more somatic complaints, more suicidal symptoms, and were more frequently diagnosed with Major Depression, compared to children without PTSD. The latter result is in line with a number of studies reporting depression to be a frequent comorbid disorder among traumatized refugee children [43] as well as among children in tsunami-affected areas [44].

Limitations of the present study are the relatively small sample size and the fact that, due to the unsafe situation and political restrictions given at that time, schools could not be selected on a random basis. However, even without a large sample representing the entire North East of the country we could obtain enough statistical power to answer our initial key question regarding the predictors and effect of traumatization in children living in a conflict region. Interviews with parents could not be obtained in the present study due to logistical reasons. Ideally, future investigations will utilize a design which allows for information on substance use and family violence to be obtained from parents as well as children. Interviews with both parents and their children would also be needed to investigate the hypothesized link between war exposure, increased use of alcohol or other substances, and an increased level of abusive behavior towards children. The present findings should be seen as a first step towards defining a relationship between war and family violence and therefore stimulate more research in the area of indirect consequences of war and organized violence on children with a particular focus on family violence.

## Conclusion

Based on the present findings, it can be asserted that children in post-war societies might not only be affected by aversive events occurring within the framework of organized violence, but also by family-related stressors, such as violence at home and parental substance use. A particularly important outcome was the association between previous war violence and the amount of violent behavior inflicted on children in their families. The high rate of PTSD found in the children was strongly linked to the amount of exposure to war, natural disaster, and family violence, thus highlighting the extremely detrimental effect that the experience of cumulative stress can have on children's mental health.

Findings of the present study point towards an urgent need for targeted interventions. Psychological treatment approaches for children in post-conflict settings should be carefully tailored to fit the specific requirements of the given population and address not only the war trauma but also psychosocial problems present at the family and community level which arise as consequences of the violent conflict. Such approaches could include trauma-focused treatment modules for the children as well as programs of stress management for the entire family that might have a beneficial effect on parental discipline method.

## Competing interests

The authors declare that they have no competing interests.

## Authors' contributions

CC conceived the study, developed the design, trained the local counselors, supervised data acquisition, performed the statistical analysis and drafted the manuscript. NJ trained and supervised the local counselors, coordinated school interviews and entered data. ES trained the local counselors and participated in initial coordination of the survey. MK trained and supervised the local counselors, identified schools, and organized the interviews with children. FN participated in the study design, analysis of data and manuscript preparation. All authors read and approved the final manuscript.

## Pre-publication history

The pre-publication history for this paper can be accessed here:


